# Cobalt Nanoparticles/Black Phosphorus Nanosheets: An Efficient Catalyst for Electrochemical Oxygen Evolution

**DOI:** 10.1002/advs.201800575

**Published:** 2018-06-10

**Authors:** Fangbing Shi, Zhibin Geng, Keke Huang, Qingshuang Liang, Yuan Zhang, Yu Sun, Jungang Cao, Shouhua Feng

**Affiliations:** ^1^ State Key Laboratory of Inorganic Synthesis and Preparative Chemistry College of Chemistry Jilin University Changchun 130012 P. R. China; ^2^ College of Chemistry and Material Fujian Normal University Fuzhou Fujian 350007 P. R. China

**Keywords:** black phosphorus, cobalt nanoparticles, electrons transfer, oxygen evolution reaction

## Abstract

Black phosphorus (BP) nanosheet (NS) is an emerging oxygen evolution reaction (OER) electrocatalyst with both high conductivity and abundant active sites. However, its ultrathin structure suffers instability because of the lone pair electrons exposed at the surface, which badly restricts durability for achieving long‐term OER catalysis. Herein, a facile solvothermal reduction route is designed to fabricate Co/BP NSs hybrid electrocatalyst by in situ growth of cobalt nanoparticles on BP NSs. Notably, electronic structure engineering of Co/BP NSs catalyst is observed by electron migration from BP to Co due to the higher Fermi level of BP than that of Co. Because of the preferential migration of the active lone pairs from the defect of BP NSs, the stability and high hole mobility can be effectively retained. Consequently, Co/BP NSs electrocatalyst exhibits outstanding OER performance, with an overpotential of 310 mV at 10 mA cm^−2^, and excellent stability in alkaline media, indicating the potential for the alternatives of commercial IrO_2_. This study provides insightful understanding into engineering electronic structure of BP NSs by fully utilizing defect and provides a new idea to design hybrid electrocatalysts.

Electrochemical water splitting is considered as a prospective solution to provide clean and sustainable energy storage instead of fossil fuels such as coal, petroleum, and natural gas.^1^ However, the half‐cell reaction in water splitting, oxygen evolution reaction (OER) via a four electron‐transfer reaction of 4OH^−^→2H_2_O+O_2_+4e^−^, demands for a high overpotential to overcome the kinetic barrier to achieve the desired current density. As a result, the widespread application of water splitting is largely hindered. Some precious metal oxides such as IrO_2_ and RuO_2_ have been reported to exhibit relatively low OER overpotentials, while the scarcity and high cost greatly limit them in large‐scale practical production.^2^ As such, tremendous efforts have been paid to search for high‐performance alternatives with low‐cost and earth‐abundant merits, including many carbon‐based materials, transition metals and their oxides/hydroxide.^3^ Nevertheless, further constructing efficient OER electrocatalysts is still highly challenging to cope with the sluggish proton‐coupled electron transfer.

Since early 2014, black phosphorus (BP) has attracted soaring interest as an emerging 2D semiconductor material, and thus has given rise to extensive researches and applications in transistors, photocatalysis, batteries, gas sensors, and photothermal therapy, etc.^4^ However, relatively little has been reported on BP‐based catalysts for OER performance, though bulk BP has previously demonstrated potential to electrocatalyze OER.^5^ Until very recently, BP nanosheets (NSs) as promising OER electrocatalysts have just been implemented and have exhibited good OER activities, owing to its high electronic conductivity and abundant surface active sites generated from the ultrathin 2D structure.^6^ Whereas, the ultrathin BP NSs structure is extremely limited by stability; due to the existence of exposed lone pairs, the surface of monodispersed BP NSs suffers from fast degradation, which is the major defect preventing BP NSs from long‐term electrocatalysis.^7^ For this reason, recent strategies to protect the BP NSs are based on passivating the exposed lone pairs by either coating BP NSs with various protective materials or occupying the lone pairs with other elements; yet very few are related to take advantage of the active lone pair electrons to realize electronic structure innovation.[[qv: 5b,8]] Considering electronic structure engineering and mechanism explorations of BP NSs‐based catalysts toward electrochemical OER, it is feasible to combine BP NSs with transitional metals possessing inherently excellent electrons migration ability for further improving the OER performance.

Herein, Co/BP NSs with Co nanoparticles (NPs) in suit supported on the BP NSs is constructed to optimize their electronic structure by a facile one‐pot solvothermal reduction process. In this case, due to the higher Fermi level of BP than that of Co NPs, electrons will migrate from BP to Co through the contact interface to achieve *E*_F_ equilibrium. Such preferential migration of the active lone pair electrons by full utilization of defect, can not only effectively protects BP NSs from degradation but also leaves them with high hole mobility; subsequently, enhanced catalytic activity and stability for OER is delivered. The as‐synthesized Co/BP nanohybrids exhibit outstanding OER performance with an early onset potential of 210 mV, a low overpotential (310 mV at 10 mA cm^−2^), and a small Tafel slope (61 mV dec^−1^) as well as excellent electrocatalytic stability.

The ultrathin BP NSs were prepared by utilizing a sonication‐assisted exfoliation from the bulk BP crystal (see Figures S1–S4 and Experimental Section in the Supporting Information) in *N*‐methyl‐2‐pyrrolidone (NMP) under an ice bath. The supernatant of BP NSs dispersing in NMP was obtained after centrifugation, resulting in a golden brown dispersion (Figure S5, Supporting Information). A typical lamellar structure of the as‐exfoliated BP NSs is shown in Figure S6 (Supporting Information) and **Figure** **1**a. The high‐resolution transmission electron microscopy (HR‐TEM) of the BP NSs (Figure 1b) shows the lattice spacing of 0.257 nm, which is consistent with the (111) plane of BP crystal. And discrete spots from the selected‐area electron diffraction (SAED) (inset in Figure 1b) were observed correspond to the (022), (040), and (021) planes of the BP crystal, respectively. Based on the analysis of HR‐TEM and SAED, it can be confirmed that each BP nanosheet is crystalline.[[qv: 4c]] Besides, it should be noted that it is difficult to obtain high‐quality SAED patterns due to the electron beam would destroy the crystalline BP and change it to amorphous during data acquisition.^9^ The above analyses confirm that the as‐exfoliated BP NSs retain the original crystalline state.

**Figure 1:**
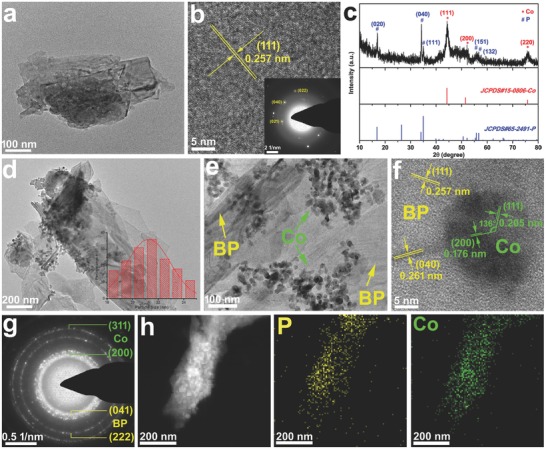
a) XRD patterns of the as‐prepared Co/BP nanohybrids. b) TEM image of the exfoliated BP nanosheets with the size of hundreds of nanometers. c) HRTEM image of the BP nanosheets (the insets are corresponding SAED patterns). d,e) TEM images of the Co/BP. f) HRTEM image and g) corresponding SAED pattern of the Co/BP. h) STEM‐EDS elemental mapping images of the Co/BP.

Typically, Co/BP NSs were prepared by reacting the as‐exfoliated BP NSs with cobalt nitrate in the presence of oleylamine (OAm) at 245 °C for 1 h, since OAm can serve as a weak reducing agent at high‐temperature to reduce Co ions,^10^ and prompt Co NPs to in situ grow on BP NSs. Comparing the X‐ray diffraction (XRD) patterns acquired from the bulk BP (Figure S2, Supporting Information) and Co/BP NSs (Figure 1c), three peaks at 44.2°, 51.6°, and 75.8° can be indexed to the (111), (200), and (220) crystal planes of cubic Co, respectively, and other peaks originate from orthorhombic BP. For Co/BP NSs, the (040) plane is still the strongest peak, revealing that the crystalline plane parallel to the layered structure is well maintained in Co/BP NSs.^11^ Scanning electron microscopy (SEM) images (Figure S7, Supporting Information) and low‐resolution TEM images (Figure 1d,e) also show that the Co/BP NSs inherits the lamellar morphology, and lots of small Co NPs with an average size of ≈21 nm are supported on the surface of BP NSs. The Co/P atomic ratio of Co/BP is close to 1: 1 as shown in the energy dispersive spectrometer (EDS) spectrum (Figure S8, Supporting Information). HR‐TEM was carried out to directly observe the interior structure of the Co/BP. As can be seen in Figure 1f, clear lattice fringes with d‐spacing of 0.205 and 0.176 nm are corresponded to the (111) and (200) crystal planes of the cubic Co, while the lattice distances of 0.261 and 0.257 nm are attributed to the (040) and (111) facets of BP. Furthermore, the SAED pattern (Figure 1g) shows that the major diffraction peaks match well with both cubic Co and orthorhombic BP. The HAADF‐scanning transmission electron microscopy (STEM) image, corresponding energy dispersive X‐ray (EDX) elemental mapping analysis (Figure 1h) clearly shows the distribution of P and Co elements, which further confirms the growth of cobalt NPs on BP NSs.

Raman scattering was performed to characterize the bulk BP, BP NSs, and Co/BP (**Figure** **2**a). The Raman spectrum of the bulk BP exhibits three characteristic peaks assigned to one out of‐plane phonon mode *A*1 g at 361.0 cm^−1^ and two in‐plane modes *B*_2g_ and *A*2 g at 436.1 and 464.0 cm^−1^, respectively.^12^ These three peaks are also observed in the Raman spectrum of BP NSs, and Co/BP, indicating that both BP NSs and Co/BP keep the unique vibrational structure of bulk BP. According to the previous work, the shifts of BP Raman peaks are layer‐dependent and with the decrease of thickness they usually shift to higher wavenumbers.^9^, ^13^ Thus, the observation of a slight movement to high wavenumber for the three peaks evidenced that the bulk BP has been exfoliated into ultrathin 2D sheets successfully. However, in contrast to these sharp peaks in the bulk BP and BP NSs, signals are apparently weakened and broadened for Co/BP, Furthermore, the *A*1 g, *B*_2g_, and *A*g 2 vibrational modes of Co/BP are blueshifted compared with that of the BP NSs, implying strong electronic interactions between Co NPs and BP NSs.^14^

**Figure 2:**
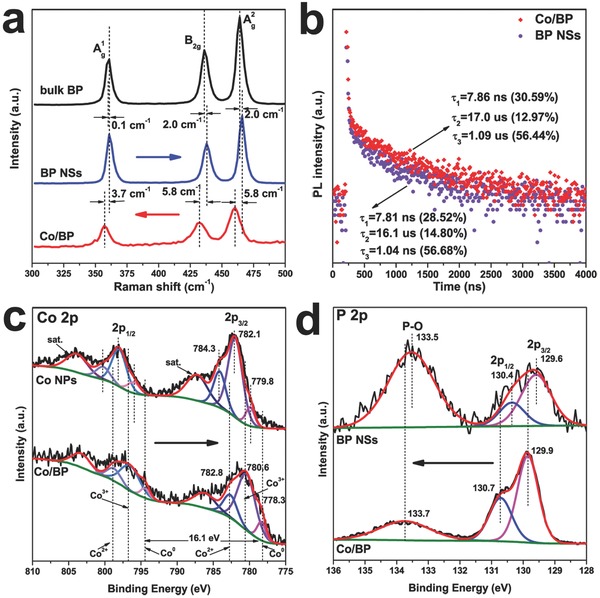
a) Raman spectra of bulk BP, exfoliated BP nanosheets, and Co/BP nanohybrids. b) The TRPL spectra of exfoliated BP nanosheets and Co/BP nanohybrids. c) Co 2p XPS spectra of Co/BP. d) P 2p XPS spectra of exfoliated BP nanosheets and Co/BP.

In order to further probe the electronic interactions and electronic states of the surface element in Co/BP, X‐ray photoelectron spectroscopy (XPS) studies were carried out. The survey spectrum (Figure S9, Supporting Information) of Co/BP indicates the presence of Co, P, C, and O elements. The high‐resolution spectrum of Co/BP (Figure 2c) in the Co 2p region shows two main peaks, along with two satellites. Co 2p_3/2_ and 2p_1/2_ peaks can be attributed to three different valences, i.e., Co^0^, Co^2+^, and Co^3+^, where Co^2+^ and Co^3+^ are derived from the unavoidable surface oxidation.^15^ The binding energies (BE) at 777.8 and 793.6 eV are assigned to Co^0^ and the splitting between the 2p_3/2_ and 2p_1/2_ is about 15.7 eV, with the full width at half maximum (FWHM) characteristically less than 1.5 eV as reported by previous work.^16^ Figure 2d shows the P 2p spectra of bare BP NSs and Co/BP. For bare BP NSs, three prominent peaks appear at 129.6, 130.5, and 133.6 eV, corresponding to P 2p_3/2_, P 2p_1/2_, and oxidized phosphorus (P‐O) BE, respectively.[[qv: 8a,17]] Notably, as shown in Figure 2c,d, these peaks exhibit a shift to lower BE by 1.5 eV for Co 2p, higher BE by 0.3 eV for P 2p and 0.2 eV for P‐O in Co/BP. The increase of BE suggests the weakened electron screening effect because of the decrease of the electron density, whereas the decrease of BE indicates an increase of electron density.^18^ Hence, in our case, it is reasonable to deduce that the lower and higher BE shifts of Co 2p and P 2p in Co/BP are attributed to the increased and decreased electron density of Co and BP, respectively, because of electrons transfer from BP to Co through the Co/BP contact interface, as will be discussed in detail later. In addition, it is noteworthy that intense P‐O sub‐bands emerged in Figure 2d for BP NSs. This phenomenon has been reported for bare BP as a result of oxidative degradation.^19^ Oppositely, only weak P‐O peak is observed for Co/BP, indicting that electrons transfer from BP to Co effectively protects BP NSs from degradation. Moreover, in Co 2p and P 2p XPS spectra, the peak corresponding to Co−P bond is not observed, which demonstrated that the van der Waals force is the driving force to enable the formation of Co/BP interfaces as indicated by FT‐IR spectroscopy (Figure S10, Supporting Information).

Theoretically, Metal‐semiconductor interface (i.e., Schottky junction) is the potential barrier between the Fermi level in the metal and the majority carrier's band edge of the semiconductor at that interface.^20^ In general, when a metal directly contacts with a semiconductor, a Schottky junction, which depends on their work functions, can be formed at the contact interface to influence the transfer of charge carriers from the semiconductor to the metal.^21^ In addition, the presence of semiconductor surface states within the bandgap between conduction and valence bands leads to band bending at the semiconductor‐vacuum interface.^20^ The ultraviolet photoelectron spectroscopy (UPS) has been conducted to understand the values of work function (difference between the Fermi and vacuum level) for BP NSs and Co NPs, which are calculated to be 3.93 and 4.42 eV (Figures S11 and S12, Supporting Information), by subtracting the width of the He I UPS spectra at excitation energy of 21.22 eV. The values of work function for BP NSs and Co NPs obtained from the UPS measurement are in good agreement with previous reports.^22^ The onset in the UPS spectrum yield corresponds to the vacuum level of the sample with respect to the work function, and thus directly yields the Fermi energy. For the vacuum synthesized BP NSs and Co NPs, the Fermi energy is −3.93 eV for BP NSs and −4.42 eV for Co NPs. Simple energy band diagram for the Co/BP hybrid material is shown in **Figure** **3**, the *E*_F_ is −3.93 eV for BP NSs higher than that of Co NPs (−4.42 eV). Hence electrons transfer from BP NSs to Co NPs will occur in Co/BP to reach *E*_F_ equilibrium, which is in agreement with the aforementioned XPS experimental results. We further analyzed the UV spectroscopy and the band gap of BP NPs and the Co/BP hybrid material. As shown in Figures S13 and S14 (Supporting Information), the bandgap of Co/BP (1.43 eV) is narrower than that of BP NPs (1.88 eV). The narrowed bandgap would greatly facilitate the charge transfer.

**Figure 3:**
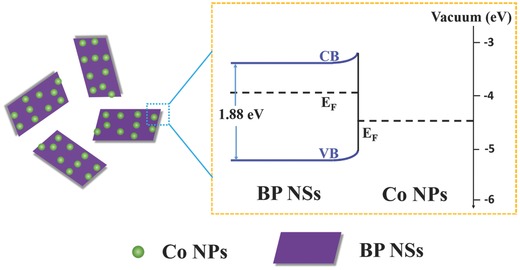
Energy band diagrams of Co/BP nanohybrids.

The steady‐state photoluminescence (PL) spectra and time‐resolved PL spectra (TRPL) were carried out under 400 nm excitation to investigate the dynamics of the charge separation and migration process in our Co/BP hybrid material. Figure S15 (Supporting Information) shows the decreased steady‐state PL intensity in Co/BP, compared to that of BP NPs, suggesting its inhibited charge recombination after coupling with Co NPs. Moreover, the TRPL (Figure 2b) presents that the short (τ1), middle (τ2), and long (τ3) PL lifetimes are extended in Co/BP compared to those of BP NPs, implying that the presence of Co NPs can effectively suppress the charge carriers recombination and elongate their lifetime. The decreased PL intensity and elongated lifetime in Co/BP evidence the better interfacial charge separation and transfer in Co/BP system.^23^

To evaluate the electrochemical activity brought by electronic structure engineering, electrocatalytic OER performance for Co/BP is evaluated by linear sweep voltammetry (LSV) in 1.0 m KOH (see the Experimental Section for details). Commercial IrO_2_, Co NPs, BP NSs, the bulk BP, and bare glassy carbon electrode (GCE) were also examined for comparison. All above samples were at the same mass loading as that of Co/BP. **Figure** **4**a shows the LSV curves after iR correction on a reversible hydrogen electrode (RHE) scale (Figure S16 shows LSV curve without iR correction, Supporting Information). The bare GCE is not active for OER; bulk BP, even bare BP NSs and Co NPs display rather poor OER performance. In contrast, Co/BP exhibits superior catalytic activity as evidenced by its earlier onset potential of ≈210 mV (Figure 4b), and even higher activity than IrO_2_ above an overpotential of 340 mV. Significantly, the overpotential at the current density of 10 mA cm^−2^, a criterion and commonly used to evaluate the OER activity,^24^ is 310 mV for Co/BP, which is merely 13 mV larger than that of freshly loaded IrO_2_ (≈297 mV). Such value in Co/BP is comparable with recently reported cobalt‐based and BP‐based OER electrocatalysts (Table S1, Supporting Information). To further explore the meliority of this novel structure, a control study was conducted by taking the physical mixture of Co NPs and BP NSs (donated as Co+BP) as a contrast experiment, which was prepared by the same chemical composition and molar ratio as the Co/BP. As can be seen in Figure 4a, the mixture of Co+BP is able to electrocatalyze OER, but its activity is not high, suggesting that the synergistic coupling between Co and BP NSs is indispensable to the high OER activity of the hybrid. In addition, when the current density is up to 50 mA cm^−2^, only a small overpotential of 360 mV is required for Co/BP, while with regard to Co NPs, BP NSs, or the Co+BP mixture, the current density cannot reach 50 mA cm^−2^, indicating that the dramatic enhancement in the OER activity for Co/BP is ascribed to the electron transfer from BP to Co through the Co/BP interface. It should be noted that during the electrochemical measurement there are two oxidation peaks in the polarization curve of Co/BP electrocatalyst at ≈1.15 and ≈1.4 V versus RHE resulting from the redox couple of Co^2+/3+^ and Co^3+/4+^ (Figure S17, Supporting Information), respectively.^15^, ^25^ As evidenced by the XRD, TEM, and XPS images (Figures S18 and S19, Supporting Information) after the OER test. A thin layer of Co_3_O_4_ was identified surrounding Co NPs, indicating that those active species (Co^2+^, Co^3+^ from Co_3_O_4_ and Co^4+^oxidized from Co^3+^) have similar OER activity as metallic Co.^25^

**Figure 4:**
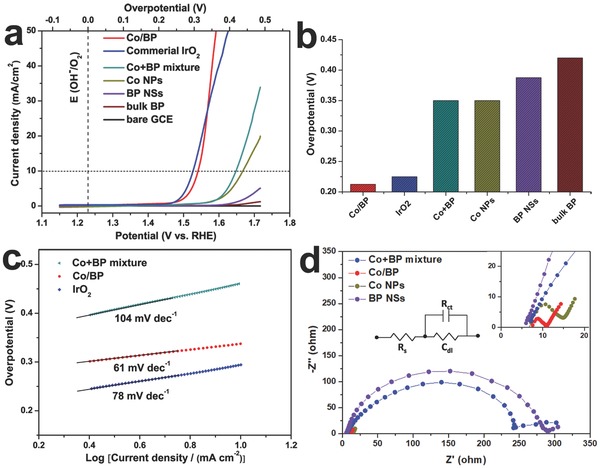
a) LSV curves and b) onset overpotential of Co/BP nanohybrids, commercial IrO_2_, the physical mixture of Co+BP, Co NPs, BP NSs, the bulk BP, and bare GCE. c) Tafel plots of Co/BP nanohybrids, commercial IrO_2_ and the physical mixture of Co+BP. d) Nyquist plots of the physical mixture of Co+BP, Co/BP nanohybrids, Co NPs and BP NSs, with inset showing the electrical equivalent circuit used to simulate the Nyquist plots .The upper right inset is the magnification of the Nyquist plots.

Tafel plots and electrochemical impedance spectra (EIS) are analyzed to get insight into the OER kinetics of the as‐prepared catalysts. Tafel slope quantitatively provides insightful information on the rate‐determining step of the OER process.^26^ In general, under alkaline solutions, oxygen evolution undergoes a multistep reaction process for converting OH^−^ to O_2_. It is accepted that the first step of OER is a process of electron transfer and if this is the rate‐determining step, the relevant Tafel slope is 120 mV dec^−1^. The second step is more complex, in which the corresponding Tafel slope is 60 mV dec^−1^ if a chemical reaction is the rate‐limiting step, while it becomes 40 mV dec^−1^ if the rate‐limiting step is an electron–proton reaction.^27^ As shown in Figure 4c, Co+BP mixture shows Tafel slope of 104 mV dec^−1^. This result reveals that the electron transfer process is the rate‐limiting step of OER in the catalyst. In contrast, Co/BP exhibited a Tafel slope of 61 mV dec^−1^. The variation indicates that the transfer of electrons is no longer the rate‐limiting step that determines the OER activity of Co/BP, and on the other hand this transition in rate‐limiting steps reveals that sufficient electrons accumulate on the Co NPs of the Co/BP‐modified electrode surface. The Nyquist plots (the corresponding equivalent circuit in the inset of Figure 4d) provide further evidence for the fast charge‐transfer of Co/BP. As shown in the upper right inset of Figure 3d, the charge‐transfer resistance (*R*_CT_) of the Co/BP is about 4.2 Ω cm^2^, which is smaller than that of BP NSs, Co NPs, and Co+BP mixture. This result reveals that the accumulated electrons on Co in the Co/BP facilitate the charge‐transfer, resulting in the superior OER kinetics. We collected the oxygen gas evolved from the OER process by using the water drainage method according to previous report,^28^ and then calculated the moles of O_2_ generated from the OER over Co/BP based on gas laws. The theoretically expected amount of O_2_ was calculated by applying the Faraday law. The theoretical and experimental amounts of O_2_ evolved from Co/BP catalyzing OER are plotted against time in Figure S20 (Supporting Information), a Faradaic efficiency of >90% is achieved.

Another important metric to evaluate the performance for OER is durability. We thus examined the stability by applying continuous cyclic voltammetry (CV) ranging from 1.22 to 1.32 V versus RHE at a scan rate of 10 mV s^−1^ at room temperature. In **Figure** **5**a, the LSV polarization curve of the Co/BP after 1000 cycles remains almost overlap and a mere 16 mV shift at a current density of 50 mA cm^−2^ is observed, confirming the excellent stability of Co/BP during the continuous CV tests. Moreover, constant potential test further evidences the electrochemical durability with maintained activity for 55 h (Figure 5b).The electrochemical active surface area (ECSA) has been evaluated by the double layer capacitance (*C*_dl_) at different scan rates in Figure 5c for Co/BP and Figure S21 (Supporting Information) for Co+BP. As shown in Figure 5d, Co/BP nanohybrids exhibit a larger *C*_dl_ (76.0 mF cm^−2^) than the mixture of Co+BP (25.6 mF cm^−2^), suggesting that more active surface area is created in Co/BP.

**Figure 5:**
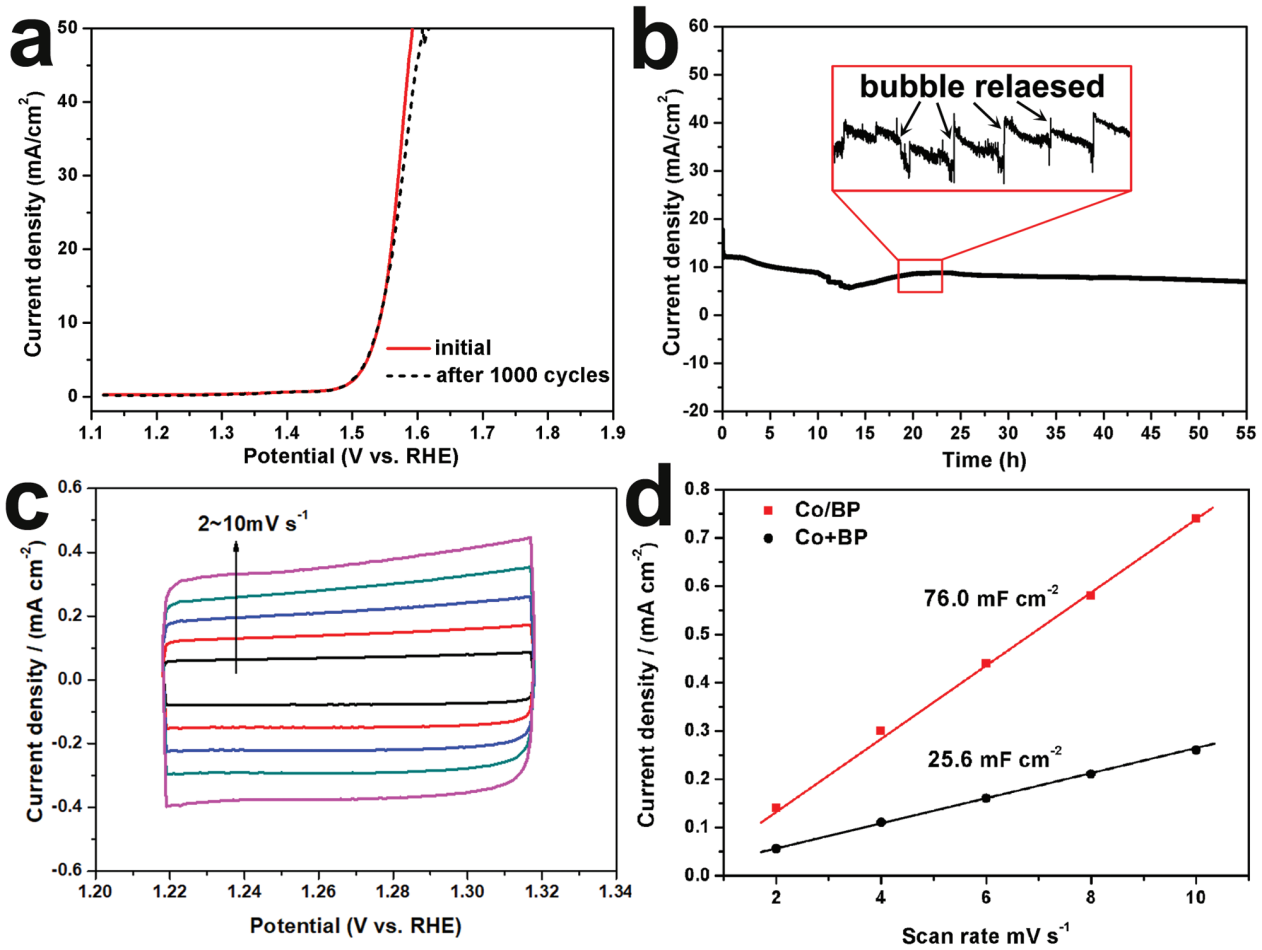
a) Stability evaluation of the Co/BP nanohybrids showing the polarization curves before and after CV cycling. b) Constant potential electrolysis of Co/BP for OER. The black arrows in the red box indicate the fluctuation caused by the bubble released. c) Cyclic voltammograms at different scan rates of Co/BP to estimate of electrochemical active surface area (ECSA). d) Current density variation plotted against scan rate fitted to a linear regression for the estimation of the double layer capacitance (*C*_dl_).

In summary, we have constructed Co/BP nanohybrids via a facile one‐pot solvothermal reduction. Through constructing the metal–semiconductor interface between Co and BP nanosheets in Co/BP nanohybrids, effective electrons migration from BP NSs to Co NPs through the contact interface is achieved due to the higher Fermi level of BP than that of metallic cobalt. Naturally, this will modify their electronic properties, lead to the electrical conductivity increase, and hence facilitate the catalytic performance for OER. The as‐prepared Co/BP nanohybrids exhibit remarkable OER activity with an overpotential of 310 mV at 10 mA cm^−2^ and a mall Tafel slope of 61 mV dec^−1^. In addition, Co/BP nanohybrids exhibited excellent electrocatalytic stability in alkaline media. The engineering and understanding of the electronic structure by forming Co/BP nanohybrids provides a straightforward strategy to design high‐efficiency and low‐cost OER electrocatalysts.

## Experimental Section

*Synthesis of Co/BP Nanohybrids*: In a typical experiment, the acquired BP nanosheets NMP dispersion (200 mL) was centrifuged at 8000 rpm for 20 min to obtain the ultrathin BP nanosheets (≈20 mg), and then the exfoliated BP nanosheets (≈20 mg) was dispersed in 15 mL oleylamine to serve as processed material to prepare Co/BP hybrid material. 0.5 mmol cobalt (II) acetate tetrahydrate (0.1245 g) and 15 mL exfoliated BP oleylamine dispersion were loaded to a 100 mL three‐neck flask attached with a Schlenk line. The mixed solution was degassed by a vacuum pump at 110 °C for 30 min to remove water and other low‐boiling impurities, with stirring and purged with Ar three times. The temperature was then rapidly raised to 245 °C and held at 245 °C for 1 h with continuous vigorous stirring. After the mixture was cooled to room temperature, 25 mL of hexane and 5 mL of ethanol were added, and the mixture was sonicated for 10 min to remove all the free ligands and the unreacted precursors. The solution was centrifuged at 8000 rpm for 10 min. The upper layer liquid was decanted, and the isolated solid was dispersed in hexane and reprecipitated by adding ethanol. The centrifugation and precipitation procedure was repeated three times and the final products were redispersed in hexane or dried under vacuum for further measurements.

*Characterizations*: Powder XRD data were collected by using a Rigaku D/Max 2550 diffractometer with a graphite monochromator using Cu‐K_α_ radiation (λ = 1.5418 Å) operating at 40 kV and 200 mA at room temperature by step scanning in the angle range of 10° ≤ 2*θ ≤* 80° with increments of 0.02°. TEM and HRTEM were recorded on a FEI Tecnai G2 S‐Twin with a field emission gun operating at 200 kV. The STEM and EDS elemental mapping images of the as‐prepared nanohybrids were determined quasiquantitatively by a Helios Nano Lab 600I from FEI Company, at an acceleration voltage of 20 kV. XPS was carried out with Thermo ESCA Lab 250 analyzer operating at constant analyzer energy mode. UV–vis absorption spectra characterized by a Shimadzu UV‐3600 spectrophotometer. Steady‐state PL spectra and TRPL spectra measurement were collected by using FLS920 from Edinburgh Instrument. Fourier Transform Infrared (FTIR) spectroscopy was recorded on NETZSCH STA499F3 QMS403D V70 from Bruker Company. High resolution UPS spectra were determined by R3000 from PREVAC sp.z.0.0.

*Electrochemical Measurements*: The as‐prepared catalysts (5 mg), Vulcan carbon black (VB, 1 mg), and Nafion solution (80 µL, 5 wt%) were dissolved in 0.5 mL 2‐propanol and sonicated for 30 min to form a slurry. Then 7.5 µL of the slurry was loaded onto the surface of a GCE (5 mm in diameter) and the electrode was dried at room temperature. The electrochemical measurements were carried out using a CHI 802D electrochemical workstation (CH Instruments, Inc., Shanghai) in a standard three‐electrode setup. A saturated calomel electrode (SCE) was used as reference electrode and a Pt electrode as counter electrode. The electrocatalytic activities of the samples toward OER were examined by obtaining polarization curves using LSV with a scan rate of 1 mV s^−1^ at room temperature in 1.0 m KOH solution using a typical three‐electrode setup with the loading of 0.33 mg cm^−2^ on a glassy carbon electrode. The stability tests of the Co/BP was performed by potential cycling from 0.2 to 0.3 V (vs RHE) at a sweep rate of 100 mV s^−1^ in 1.0 m KOH for 1000 cycles, then linear sweep voltammetry polarization curves were obtained. All current density versus potential data plots are IR corrected to compensate for all ohmic losses throughout the system, which were caused by the effect of solution resistance. IR correction was manually corrected by 90%*R*_s_ before linear sweep voltammetry, and *R*_s_ is the uncompensated resistance measured in 1 m KOH. All the potentials reported in this paper were converted to the RHE.

## Conflict of Interest

The authors declare no conflict of interest.

## Supporting information

Supplementary
