# Supplementary material for: Cobalt Nanoparticles/Black Phosphorus Nanosheets: An Efficient Catalyst for Electrochemical Oxygen Evolution
Source: Adv Sci (Weinh). 2018 Jun 10;5(8):1800575. doi: 10.1002/advs.201800575 (PMC6096989; doi:10.1002/advs.201800575)
Supplement: Supplementary file 1 — Supplementary [file ADVS-5-1800575-s001.pdf]

Copyright WILEY-VCH Verlag GmbH & Co. KGaA, 69469 Weinheim, Germany, 2018.

ADVANCED SCIENCE
Open Access

Supporting Information

for *Adv. Sci.,* DOI: 10.1002/advs.201800575

Cobalt Nanoparticles/Black Phosphorus Nanosheets: An Efficient Catalyst for Electrochemical Oxygen Evolution

*Fangbing Shi, Zhibin Geng, Keke Huang, Qingshuang Liang, Yuan Zhang, Yu Sun, Jungang Cao, and Shouhua Feng**

Copyright WILEY-VCH Verlag GmbH & Co. KGaA, 69469 Weinheim, Germany, 2016.

Supporting Information

Metallic Cobalt/Black Phosphorus: an Efficient Catalyst for Electrochemical Oxygen Evolution

*Fangbing Shi, Zhibin Geng, Keke Huang, Qingshuang Liang, Zhang Yuan, Yu Sun, Jungang Cao, Shouhua Feng**

1. Experimental Section

Materials: Red phosphorus was purchased from Sinopharm Chemical Reagent Co., Ltd. Oleylamine (OAm, 80–90%) was purchased from Aladdin. Ethanol (analytical reagent), isopropyl alcohol (analytical reagent), tin powder and iodine and cobalt (II) acetate tetrahydrate ($Co(OAc)_2$-$4H_2O$, 98%) were purchased from Beijing chemical works. Nafion was purchased from Sigma-Aldrich.

Synthesis of bulk BP: The bulk BP were obtained by a facile chemical vapor transport deposition route.[1] As illustrated in Figure S1, the bulk BP is layered crystal with metallic luster. Three sharp (020), (040), and (060) X-ray diffraction (XRD) peaks in Figure S2 reveal its fine crystal quality. The scanning electron microscopy (SEM) images clearly show the layered structure of the bulk BP crystals, where the existence of P, Sn (catalyst for BP preparation) and O (originated from the background) elements is confirmed by energy-dispersive X-ray spectroscopy (EDX) spectrum (Figure S4).

Synthesis of BP nanosheets: The BP nanosheets were prepared by using a simple liquid exfoliation technique. 7 mg bulk BP was added into 20 mL N-methyl-2-pyrrolidone (NMP) solvent. The dispersion was sonicated for 8 h under ice cooling. Subsequently, the dark brown suspension was centrifuged at 2000 rpm for 10 min to remove non-exfoliated bulk BP. The

supernatant was collected to obtain the ultrathin BP nanosheets, and then the acquired BP nanosheets NMP dispersion (20 ml) was centrifuged at 8000 rpm for 20 min. The upper layer liquid was decanted, and the isolated solid was dried under vacuum to obtain the ultrathin BP nanosheets (~2mg).

2. Figures, Tables and related discussion

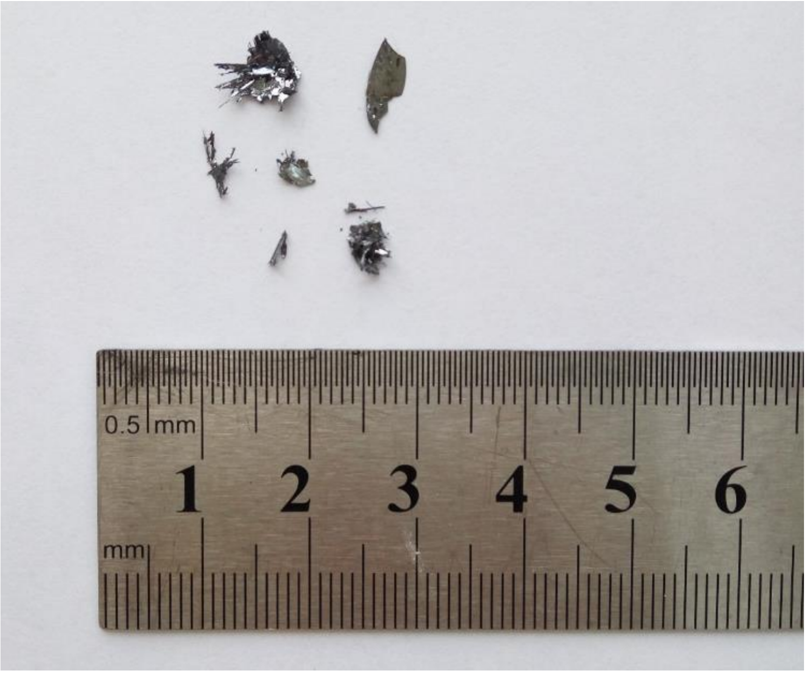

Figure S1. Photograph of the as-synthesized bulk BP.

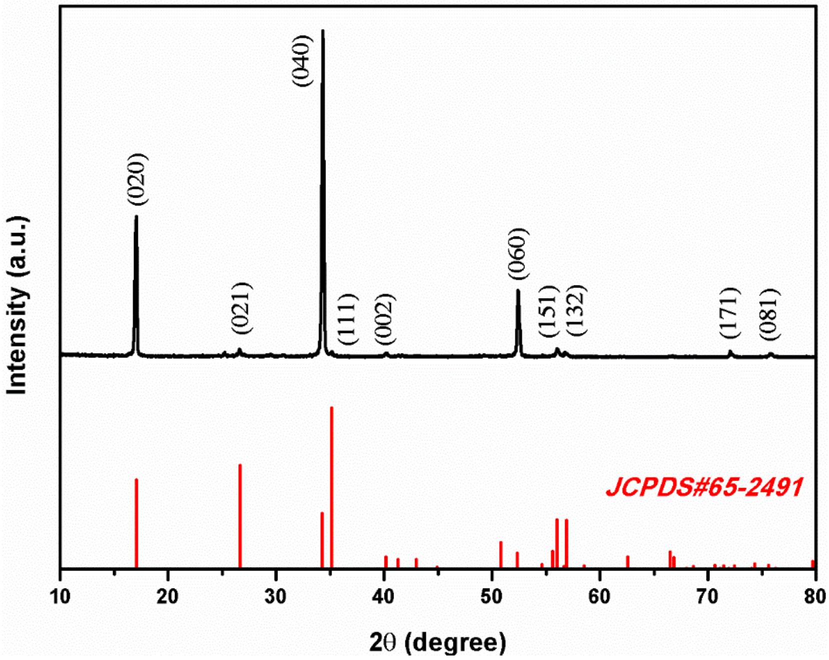

Figure S2. XRD pattern of the bulk BP compared to the standard X-Ray diffraction pattern of orthorhombic BP (JCPDS 65-2491).

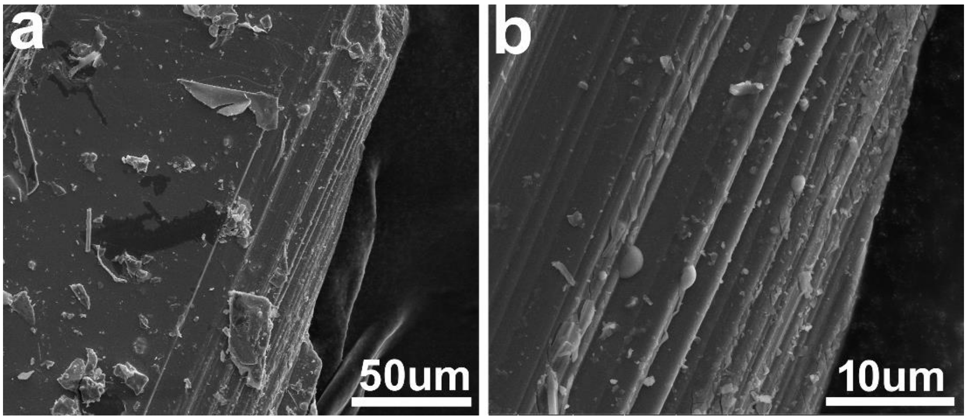

Figure S3. SEM images (a, b) of the bulk BP.

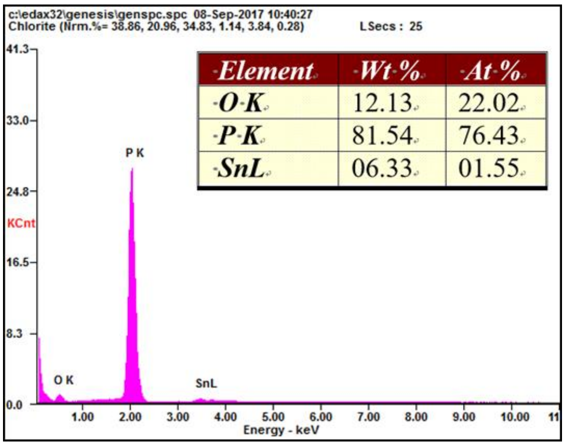

Figure S4. EDX spectrum of the bulk BP.

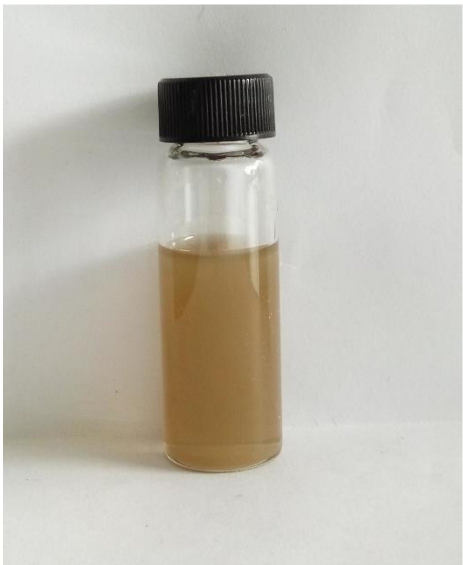

Figure S5. The supernatant of BP NSs dispersing in NMP obtained after centrifugation.

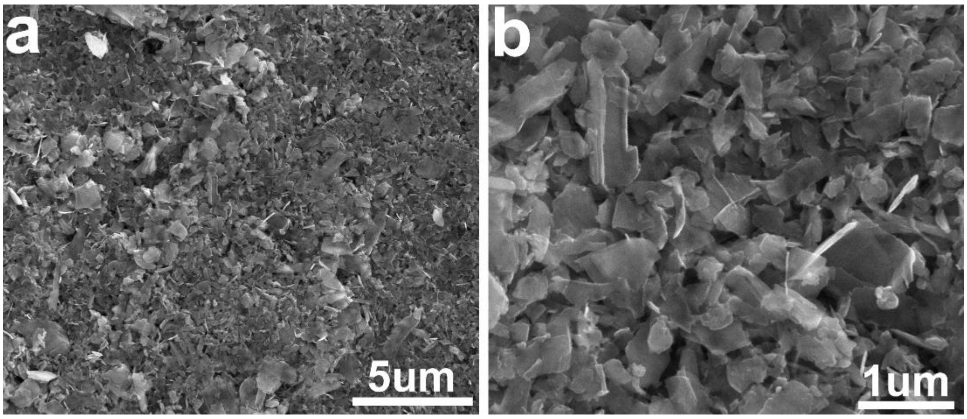

Figure S6. SEM images (a, b) of the as-exfoliated BP nanosheets (BP NSs).

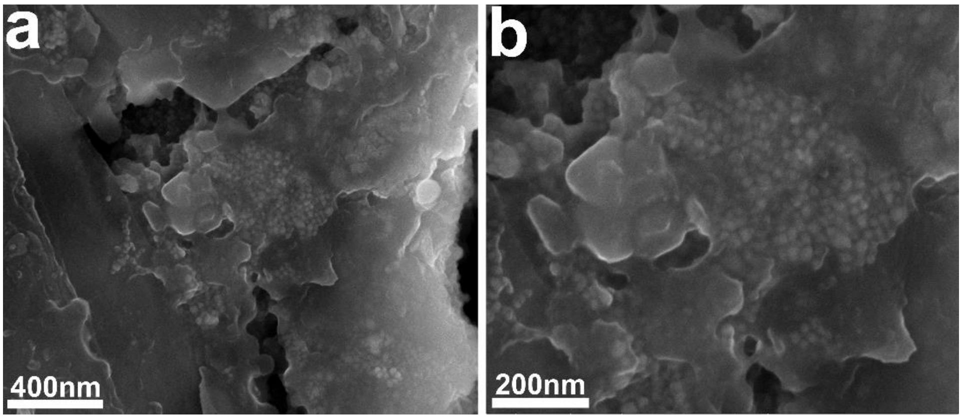

Figure S7. SEM images (a, b) of the as-prepared Co/BP nanohybrids.

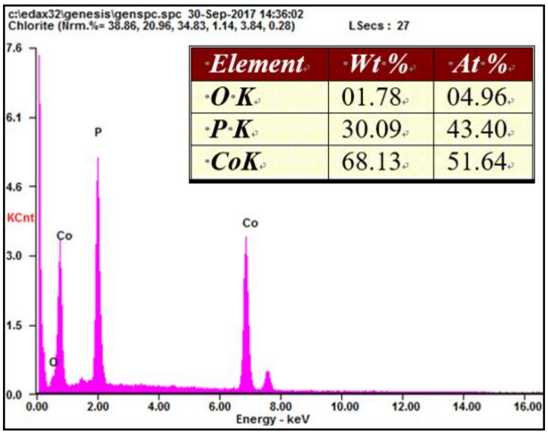

Figure S8. EDX spectrum of the the as-prepared Co/BP nanohybrids.

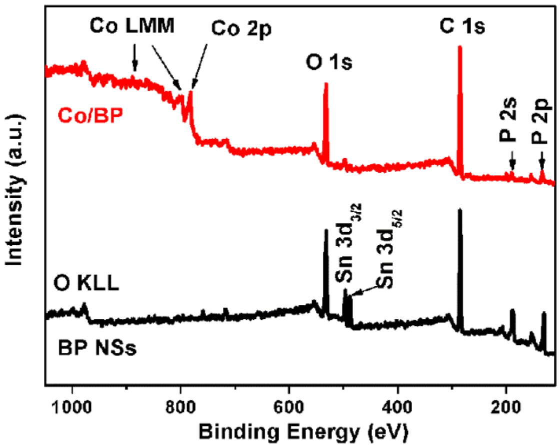

Figure S9. XPS survey spectrum of the as-prepared Co/BP nanohybrids and the as-exfoliated BP nanosheets (BP NSs). In the bare BP NSs XPS spectra by contrast, Co is not detected, while P, C, O and Sn (catalyst for BP preparation) can be detected.

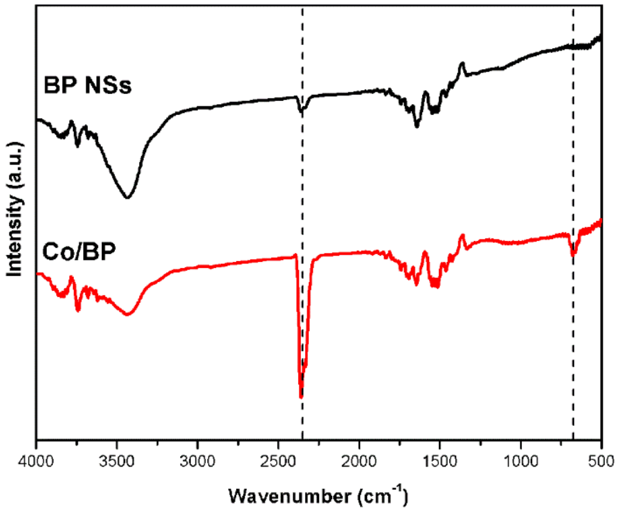

Figure S10. FT-IR spectra of BP nanosheets and Co/BP hybrid material. the absorption wavenumbers of the BP NSs centered at ~1000 cm^{-1} and ~1620 cm^{-1} can be assigned to the P–O stretching and P=O stretching mod,[2] respectively. When Co NPs were hybridized with BP, all the characteristic vibrational peaks of BP were maintained, while a strong peak located at around 2350 cm^{-1} was observed due to the bending vibration of environmental CO_2 adsorbed on the surface. The peak in Co/BP located at around 670 cm^{-1} can be assigned to the Co–O stretching mode, suggesting the unavoidable surface oxidation.[3]

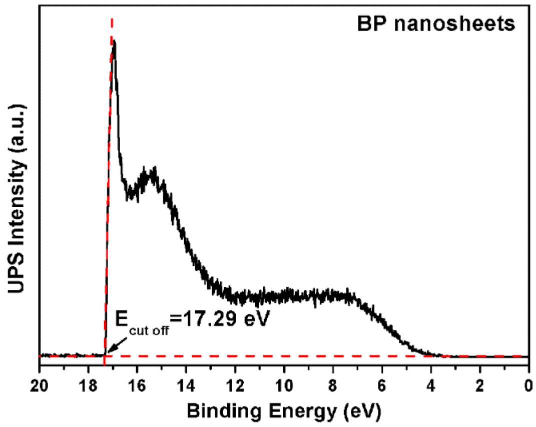

Figure S11. Ultraviolet photoelectron spectroscopy (UPS) spectrum of BP NSs. The the value of work function for BP NSs is calculated to be 3.93 eV.

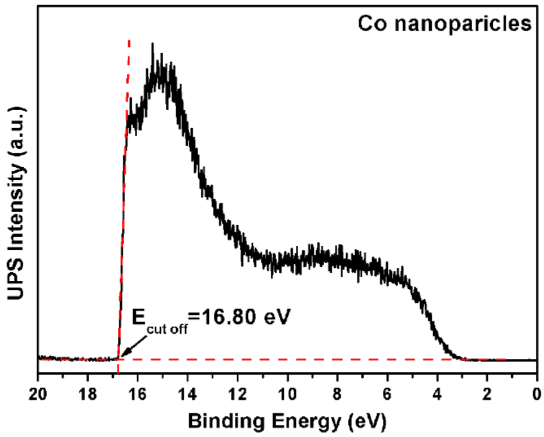

Figure S12. Ultraviolet photoelectron spectroscopy (UPS) spectrum of Co NPs. The the value of work function for Co NPs is calculated to be 4.42 eV.

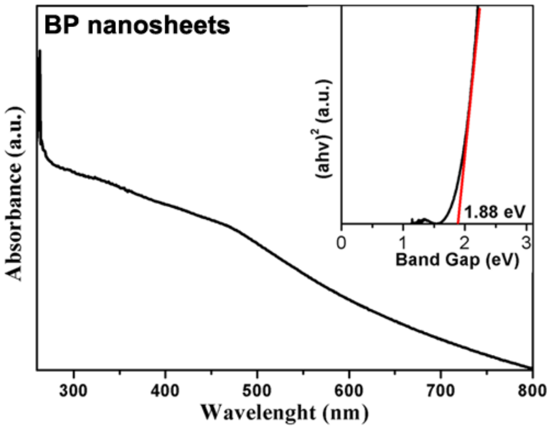

Figure S13. UV absorption spectra of BP NSs. The inset is the corresponding band gap of BP NSs.

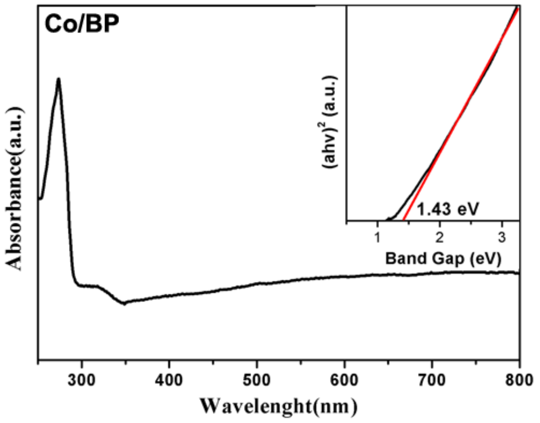

Figure S14. UV absorption spectra of Co/BP. The inset is the corresponding band gap of Co/BP.

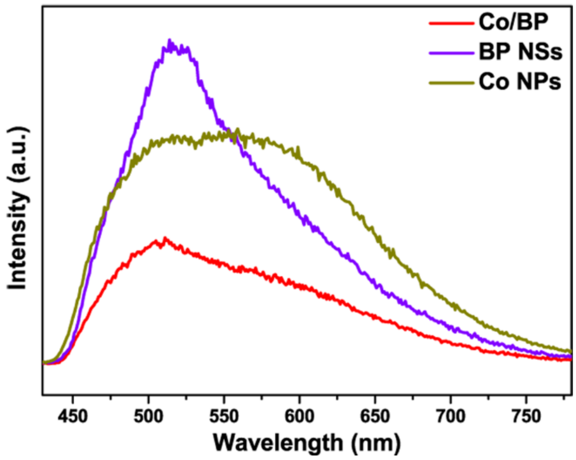

Figure S15. Steady-state photoluminescence (PL) spectra of BP NSs, Co NPs and Co/BP.

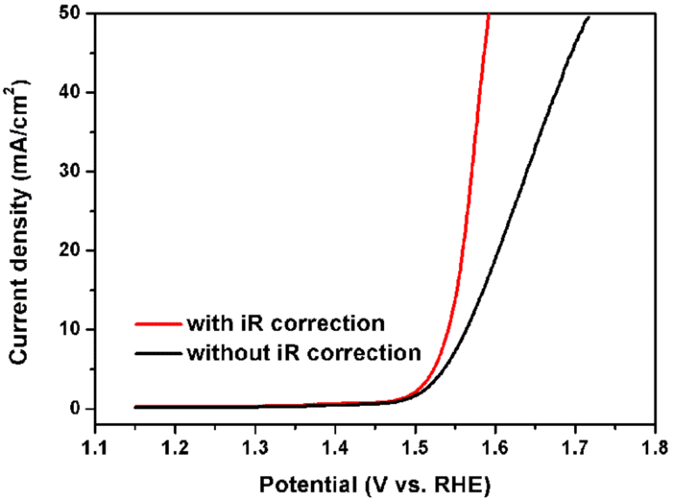

Figure S16. Polarization curves of the Co/BP electrocatalyst with and without *iR* correction.

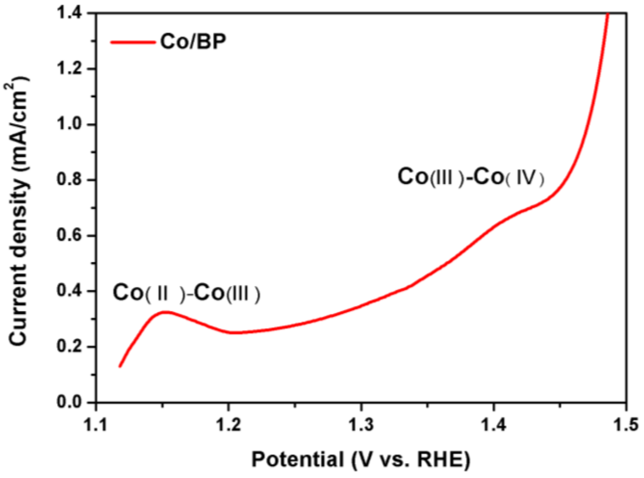

Figure S17. Magnified polarization curve of Co/BP to demonstrate the oxidation of metallic Co nanoparticles during the process of OER test.

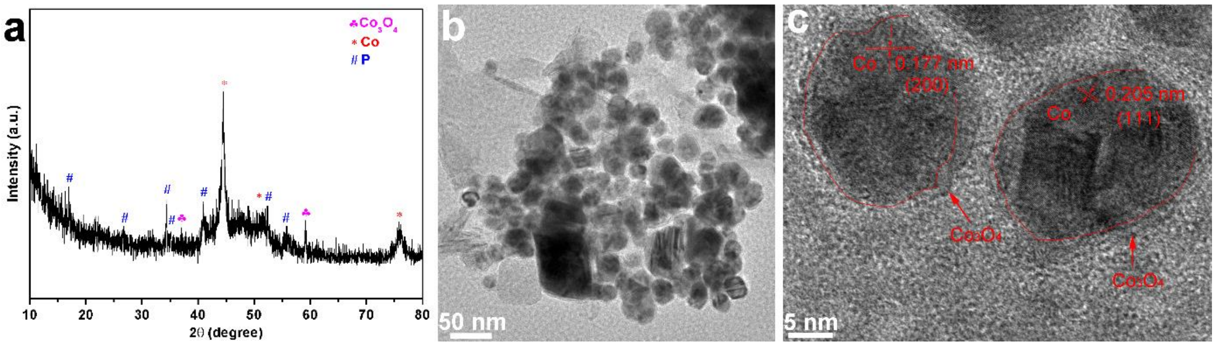

Figure S18. XRD spectrum (a), TEM image (b) and HR-TEM (c) of Co/BP electrocatalyst after the OER test.

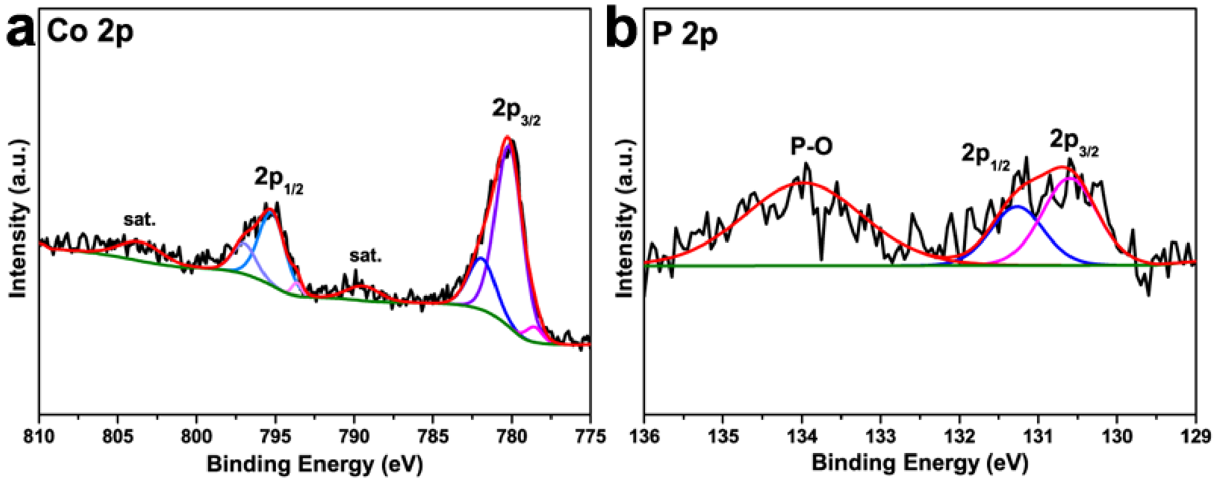

Figure S19. XPS spectrum of Co (a) and P (b) for Co/BP electrocatalyst after the OER test.

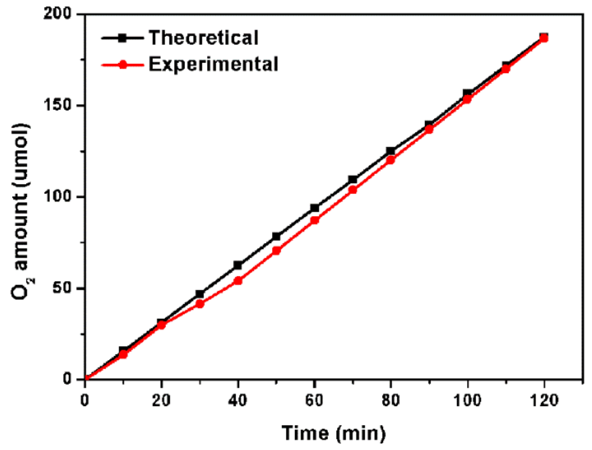

Figure S20. The amounts of O_2 theoretically calculated and experimentally measured versus time during OER.

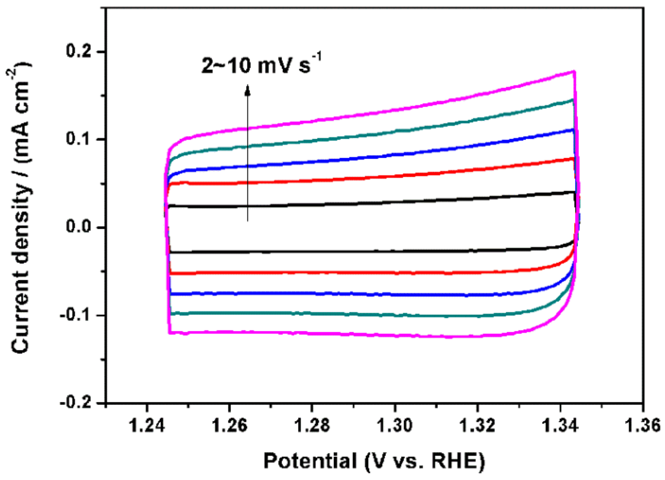

Figure S21. Cyclic voltammograms at different scan rates of the physical mixture of Co+BP to estimate of electrochemical active surface area (ECSA).

Table S1. Comparison of the OER activity of Co/BP with Co-based and BP-based electrocatalysts in literatures.

Samples	Onset potential (mV)	Overpotential at 10 mA cm^{-2} (mV)	Electrolyte	Tafel slope (mV dec^{-1})	References
Co/BP	210	310	1 M KOH	61	This work
Co_2P/BP	~250	~390	1 M KOH	78	[4]
BP NSs	230	---	1 M KOH	88	[5]
ε-Co NPs	---	473	1 M KOH	---	[6]
Co/VN	~250	320	1 M KOH	55	[7]

N-CG-CoO	340	340	1 M KOH	71	[8]
Co-CN SS	290	340	1 M KOH	78	[9]
Co_3O_4/N-rmGO	~260	310	0.1 M KOH	67	[10]
Co-NPs	---	390	0.1 M KOH	---	[11]
Co/N-C-800	---	371	0.1 M KOH	61.4	[12]
3D BP-CNT	250	370	0.1 M KOH	72.88	[13]

References

[1] M. Zhao, H. Qian, X. Niu, W. Wang, L. Guan, J. Sha, Y. Wang, *Cryst.Growth Des.* **2016**, 16, 1096.

[2] M. Ceppatelli, S. Fanetti, R. Bini, *J. Phys. Chem. C* **2013**, 117, 13129.

[3] C.-W. Tang, C.-B. Wang, S.-H. Chien, *Thermochim. Acta* **2008**, 473, 68.

[4] W. Lei, G. Liu, J. Zhang, M. Liu, *Chem. Soc. Rev.* **2017**, 46, 3492.

[5] X. Ren, J. Zhou, X. Qi, Y. Liu, Z. Huang, Z. Li, Y. Ge, S. C. Dhanabalan, J. S. Ponraj, S. Wang, J. Zhong, H. Zhang, *Adv. Eng. Mater.* **2017**, 7, 1700396.

[6] N. H. Chou, P. N. Ross, A. T. Bell, T. D. Tilley, *ChemSusChem* **2011**, 4, 1566.

[7] X. Peng, L. Wang, L. Hu, Y. Li, B. Gao, H. Song, C. Huang, X. Zhang, J. Fu, K. Huo, P. K. Chu, *Nano Energy* **2017**, 34, 1.

[8] S. Mao, Z. Wen, T. Huang, Y. Hou, J. Chen, *Energy Environ. Sci.* **2014**, 7, 609.

[9] Y. Shi, Y. Wang, Y. Yu, Z. Niu, B. Zhang, *J. Mater. Chem. A* **2017**, 5, 8897.

[10] Y. Liang, Y. Li, H. Wang, J. Zhou, J. Wang, T. Regier, H. Dai, *Nat Mater* **2011**, 10, 780.

[11] L. Wu, Q. Li, C. H. Wu, H. Zhu, A. Mendoza-Garcia, B. Shen, J. Guo, S. Sun, *J. Mater. Chem. A* **2015**, 137, 7071.

[12] Y. Su, Y. Zhu, H. Jiang, J. Shen, X. Yang, W. Zou, J. Chen, C. Li, *Nanoscale* **2014**, 6, 15080.

[13] Q. Jiang, L. Xu, N. Chen, H. Zhang, L. Dai, S. Wang, *Angew. Chem. Int. Ed.* **2016**, 55, 13849.
